# Roles of extracellular vesicles from different origins in metabolic-associated fatty liver disease: progress and perspectives

**DOI:** 10.3389/fimmu.2025.1544012

**Published:** 2025-03-10

**Authors:** Jing Wang, Shuoqiang Bao, Qi An, Caihong Li, Juan Feng

**Affiliations:** ^1^ School of Public Health, Gansu University of Chinese Medicine, Lanzhou, China; ^2^ College of Health Science and Environmental Engineering, Shenzhen Technology University, Shenzhen, China

**Keywords:** metabolic-associated fatty liver disease (MAFLD), extracellular vesicles (EVs), host-derived EVs, plant-derived EVs, microbial-derived EVs, biomarkers, therapeutic agents, therapeutic targets

## Abstract

Metabolic-Associated Fatty Liver Disease (MAFLD) is the most common chronic liver disease worldwide, associated with systemic metabolic dysregulation. It can progress from simple hepatic steatosis (MAFL) to more severe conditions like Metabolic-Associated Steatohepatitis (MASH), fibrosis, cirrhosis, and Hepatocellular Carcinoma (HCC). There is a critical lack of reliable non-invasive diagnostic methods and effective pharmaceutical treatments for MAFLD/MASH, emphasizing the need for further research. Extracellular vesicles (EVs) are nanoscale structures that play important roles in cell signaling by delivering bioactive molecules. However, there is a significant gap in literature regarding the roles of EVs from hosts, plants, and microbiota in MAFLD. This review explores the potential of EVs from various sources—host, plants, and microbiota—as biomarkers, therapeutic agents, drug carriers, and treatment targets for MAFLD. Firstly, the roles of host-derived extracellular vesicles (EVs) in MAFLD, with a focus on cell-type specific EVs and their components—proteins, miRNAs, and lipids—for disease diagnosis and monitoring were discussed. Moreover, it highlighted the therapeutic potential of mesenchymal stem cell (MSC)-derived EVs in reducing lipid accumulation and liver injury, and immune cell-derived EVs in mitigating inflammation and fibrosis. The review also discussed the use of host-derived EVs as drug carriers and therapeutic targets due to their ability to deliver bioactive molecules that impact disease mechanisms. Additionally, it summarized research on plant-derived EVs, which help reduce liver lipid accumulation, inflammation, and enhance gut barrier function in MAFLD. Also, the review explored microbial-derived EVs as novel therapeutic targets, particularly in relation to insulin resistance, liver inflammation, and dysfunction in MAFLD. Overall, by exploring the diverse roles of EVs from host, plant, and microbiota sources in MAFLD, this review offers valuable insights into their potential as non-invasive biomarkers and novel therapeutic strategies, which could pave the way for more effective diagnostic and treatment options for this increasingly prevalent liver disease. Notably, the challenges of translating EVs into clinical practice were also thoroughly discussed, aiming to provide possible directions and strategies for future research.

## Background

Non-Alcoholic Fatty Liver Disease (NAFLD) is the leading cause of chronic liver conditions worldwide, impacting ~30% of the population and with a rising prevalence (1). The disease is primarily characterized by macrovesicular steatosis in ≥ 5% of hepatocytes without any secondary causes like alcohol or drug usage (2). Due to the link between NAFLD and systemic metabolic dysregulation, it has recently been proposed to rename NAFLD as Metabolic-Associated Fatty Liver Disease (MAFLD) (3). Notably, MAFLD could advance from hepatic steatosis (fatty liver) alone, referred to as MAFL, to Metabolic-Associated Steatohepatitis (MASH), a more severe inflammatory phase marked by liver cell injury with or without fibrosis, which could, in turn, evolve into liver fibrosis, cirrhosis, and Hepatocellular Carcinoma (HCC) (4).

The exact mechanisms underlying the pathogenesis of MAFLD remain incompletely understood. MAFLD primarily arises from nutritional excess, leading to the expansion of fat depots and ectopic fat accumulation (5). This process is exacerbated by macrophage infiltration into visceral adipose tissue, which fosters a pro-inflammatory environment and promotes insulin resistance (5)—a critical mechanism in MAFLD’s pathogenesis (6, 7). Insulin resistance disrupts normal lipolysis, resulting in increased fatty acid influx to the liver (7). This, combined with augmented *de novo* lipogenesis, exceeds the liver’s metabolic capacity, precipitating an imbalance in lipid metabolism that generates lipotoxic lipids (8, 9). Such lipotoxicity induces cellular stress, characterized by oxidative and endoplasmic reticulum (ER) stress, activates inflammasomes, and leads to apoptotic cell death (8, 9). This inflammatory cascade not only drives hepatocyte injury but also stimulates tissue regeneration and fibrosis (8, 9). Pro-inflammatory and profibrotic macrophages are particularly implicated in the pathogenesis of liver fibrosis (10). Furthermore, the intricate interplay of environmental and genetic factors plays a crucial role in MAFLD, with dietary influences being especially significant (11, 12). Current research has underscored the pivotal involvement of gut microbiota in the disease’s development (13, 14). Collectively, these insights reinforce the “multiple-hit hypothesis” of MAFLD, wherein a synergistic combination of factors contributes to its onset and progression toward more severe manifestations, such as MASH (5, 15, 16).

The primary treatment for MAFLD remains lifestyle modification aimed at achieving weight loss (17). This includes regular physical activity and dietary changes (18), such as adherence to a Mediterranean diet (19). However, many individuals find it challenging to sustain an exercise regimen, and cultural preferences may hinder acceptance of the Mediterranean diet (20). Bariatric surgery offers an alternative for managing MAFLD, particularly in cases of severe obesity (20), while liver transplantation remains a critical option for advanced stages of the disease (21). Nonetheless, these surgical interventions are associated with significant risks and potential complications (20, 21). Pharmacological therapies have emerged as promising alternatives. Agents such as statins, peroxisome proliferator-activated receptor (PPAR) agonists, farnesoid X receptor (FXR) agonists, glucagon-like peptide-1 receptor (GLP-1R) agonists, and sodium-glucose cotransporter-2 (SGLT2) inhibitors have shown potential benefits in treating MAFLD (20, 22). However, their use is often complicated by different adverse effects, and large-scale clinical trial data supporting their efficacy and long-term safety in MAFLD and MASH remains limited (20, 23, 24). Resmetirom is a thyroid hormone receptor-beta (THR-β) agonist that was licensed by the U.S. Food and Drug Administration in March 2024 exclusively for the treatment of MASH (25). While it presents a novel therapeutic option, Resmetirom is also associated with several side effects (26). Consequently, the quest for safe and effective pharmacological therapies for MAFLD continues to be a pressing challenge.

MAFL can be diagnosed through a combination of imaging techniques and clinical characteristics, including the presence of metabolic comorbidities and abnormal laboratory findings. However, imaging cannot diagnose MASH; they can only help assess structural alterations in the liver and the extent of fibrosis. Currently, liver biopsy remains the “gold standard” for diagnosing MASH and assessing disease progression, including the degree of liver fibrosis (27, 28). Nevertheless, this histological evaluation carries inherent risks, including bleeding and, in rare instances, mortality (27, 28). Furthermore, histopathological assessments can be influenced by the limited representativeness of sampling sites and variability in interpretation among pathologists (27, 28). Additionally, liver biopsy presents challenges for the longitudinal monitoring of liver damage over time (28). As the development of MASH therapies advances, the need for non-invasive alternatives to liver biopsy has become critical, as these methods could identify patients in need of intervention and monitor treatment responses (27–30). In this context, liquid biopsies have demonstrated significant potential (29, 30). However, there is a significant shortage of reliable non-invasive diagnostic techniques for the diagnosis and progression of MAFLD. Thus, there is a pressing need to discover new non-invasive biomarkers for the diagnosis, staging, and prognosis of MAFLD, especially for MASH.

Extracellular vesicles (EVs) are membrane-enclosed structures secreted by prokaryotic, eukaryotic, and plant cells, and their release occurs in an evolutionarily conserved manner (31). These structures are commonly found in the Extracellular Matrix (ECM), various body fluids, and cell supernatants (32). Based on their size and biological mechanisms, EVs can be categorized into three groups: exosomes (30-150 nm), microvesicles (MVs, 100-1000 nm), and apoptotic bodies (ABs, 1-5 µm) (33). Initially, EVs were considered an avenue through which ordinary cells could handle and eliminate unwanted substances and regulate normal tissue balance, or conversely, as a way for cancer cells to support tumor advancement and spread (34, 35). Presently, EVs are acknowledged to be involved in cell-to-cell communication (36). They contain proteins, DNA, mRNA, microRNAs (miRNAs), and lipids from parent cells (37), through which their mediated signals can be transmitted (31). Active intercellular communication through EV secretion is crucial for maintaining normal physiological functions (38, 39), and EV-emitted abnormal signals are also associated with many disease states (40). In this regard, multiple studies have demonstrated the significant involvement of EVs in MAFLD progression (5, 41).

**Figure 1:**
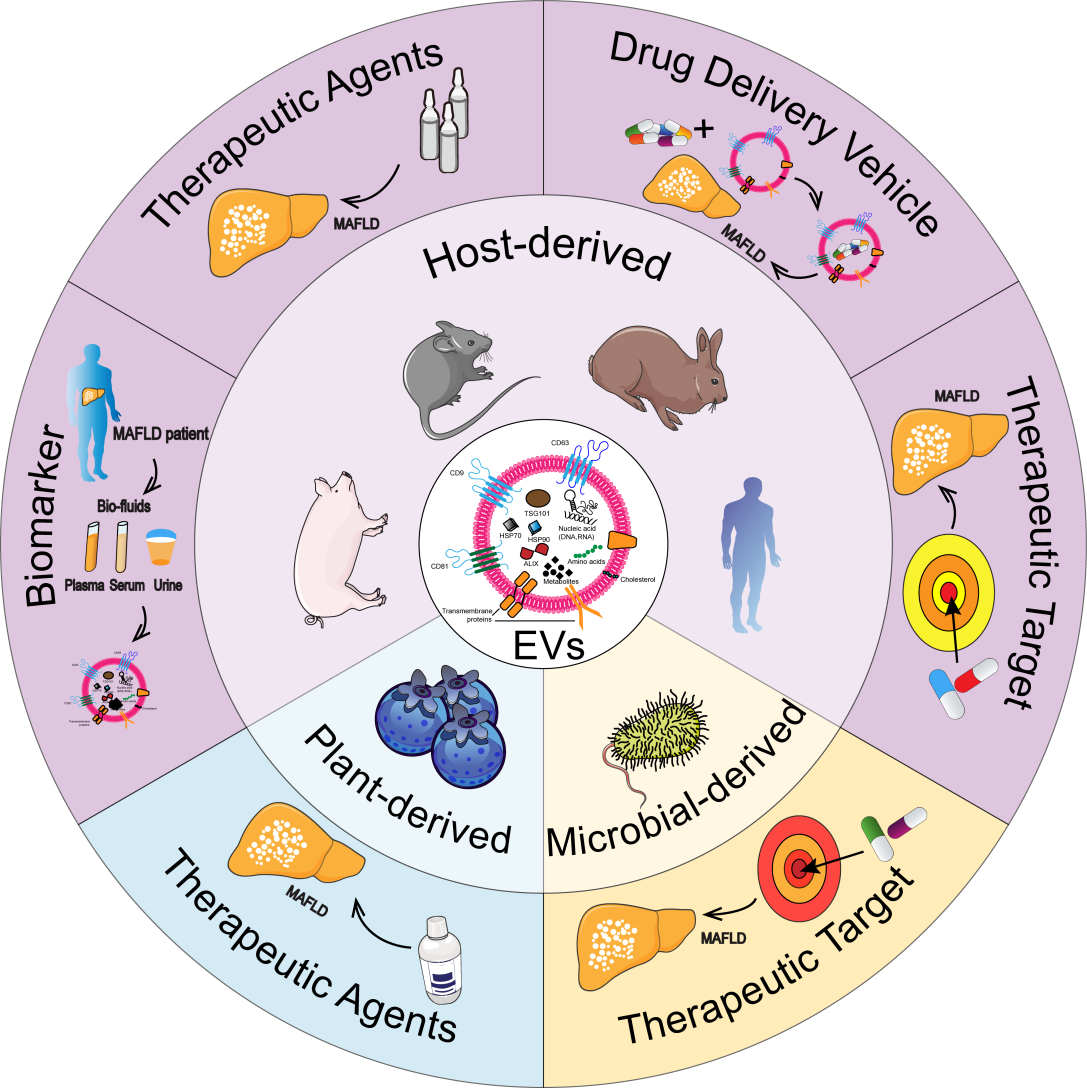
Diagram illustrating the roles of EVs from different origins in MAFLD.

This review explores the roles of EVs originating from host cells, plant cells, and microbes in MAFLD (**Figure 1**). It emphasizes the promise of host-derived EVs as non-invasive biomarkers and therapeutic targets for MAFLD diagnosis and treatment, while also considering their use as drug carriers or therapeutic agents. Furthermore, the review discusses the therapeutic prospects of plant-derived EVs and the function of microbial-derived EVs as targets in MAFLD. This comprehensive tutorial review presents, for the first time, the diverse functions of EVs from various origins in relation to MAFLD.

However, the separation, standardization, and scale-up production of EVs still face numerous challenges (42, 43). The methods for isolating EVs are diverse, including ultracentrifugation, size exclusion chromatography, reagent-based separation kits, precipitation methods, as well as immunoaffinity and microfluidic-based separation techniques (44). However, these methods lack standardized protocols, leading to significant variability in the comparability of results across different experiments (42). Furthermore, the contents of EVs, such as proteins, RNA, and lipids, are highly heterogeneous and influenced by factors such as the cell type of origin, isolation technique, and storage conditions, making the prediction of EVs’ functionality and quality challenging (42, 45). More importantly, scaling up EV production to meet clinical demand presents technical and cost-related barriers, including standardization of cell culture, yield control, and purity assurance (42–45). Addressing these issues is crucial for the widespread application of EVs as therapeutic and diagnostic tools.

## Host-derived EVs as potential biomarkers for MAFLD

### Circulating cell type‐specific EVs as potential biomarkers for MAFLD

During the disease process, the majority of cells secrete EVs into the surrounding extracellular space (46). The variations in the quantity of EVs or changes in their contents could act as disease-specific indicators and are being explored as possible blood biomarkers (47). In the case of MAFLD, both the total quantity of circulating EVs and the number of EVs derived from specific cell types, such as hepatocytes, macrophages, and neutrophils, have been found to increase with disease progression (48, 49). Using a Fat, Fructose, and Cholesterol (FFC) diet-induced MASH mouse model, Li et al. quantitatively analyzed cell-specific EVs and revealed that the concentrations of neutrophil-, macrophage-, and hepatocyte-derived EVs correlated well with MAFLD Activity Score (MAS) (49). Kornek’s team found that invariant Natural Killer T (iNKT) cell- and CD14^+^ cell-derived EVs were notably higher in the serum or plasma of MAFL/MASH patients than in healthy individuals and demonstrated clinical significance in relation to patients’ histological grading (50). Considering the close association of these cells with inflammation and fibrosis, and the fact that the pathogenesis and progression of MAFLD are characterized by liver inflammation and fibrosis, these cell type-specific extracellular vesicles may serve as specialized biomarkers, indicating the severity of inflammation in MAFL/MASH patients.

### Circulating host-derived EV proteins as potential biomarkers for MAFLD

EVs derived from host cells closely resemble their source cells, enabling them to carry a diverse array of proteins, miRNAs, and lipids that facilitate intercellular communication (51). During the transition from a healthy to a diseased state, both the quantity of circulating EVs and their cargo, such as specific proteins, can reflect the pathological and physiological status of the originating cells (52, 53). Furthermore, EVs have been identified as emerging mediators of liver injury and inflammation in the context of MAFLD (48). Consequently, the specific proteins carried by these EVs represent promising candidates for biomarkers of MAFLD. Proteomics analysis of lipotoxic EVs derived from serum samples and hepatocytes of MASH patients revealed that haptoglobin, Vascular Non-Inflammatory Molecule-1 (Vanin 1), and the Insulin-Like Growth Factor-Binding Protein Complex Acid Labile Subunit (IGF-BP ALS) could serve as biomarkers for detecting EVs originating from hepatocytes in MASH patients (54). Wnt/Frizzled receptor protein Frizzled 7 (FZD7) protein levels were upregulated in plasma exosomes derived from MAFLD patients, with lifestyle changes potentially downregulating FZD7, suggesting its promise as a new and effective biomarker for diagnosis and prognosis of MAFLD (55). Circulating levels of EpCAM^+^ CD133^+^ EVs were significantly elevated during the transition from basic steatosis to steatohepatitis, which could be used as a non-invasive diagnostic marker for hepatitis, as well as in prognosis assessment and therapy monitoring (56). Research by Povero et al., identified Solute Carrier family 27, member 5 (SLC27A5) as a distinct indicator for circulating lipotoxic hepatocyte-originating EVs in MAFLD and MASH, whereas Asialoglycoprotein Receptor 1 (ASGR1) is a specific marker for hepatocyte-derived EVs in cirrhosis, and increased with disease progression (57). In addition, EV proteins such as WISP1, AIMP1, IL27RA, ICAM2, IL1β, STK16, and RGMA can reliably differentiate healthy controls from patients with precirrhotic and cirrhotic MASH (57). Zhang’s team reported that the proportion of Glucose Transporter 1 (GLUT1)-expressing serum hepatogenic exosomes was notably higher in MASH patients than in simple MAFL patients, with higher proportions in advanced MASH stages (58). These results indicate that EV proteins hold significant promise as potential biomarkers for the diagnosis and staging of MAFLD (as summarized in **Table 1**).

**Table 1:** Summary of the reported host-derived EVs as disease markers for MAFLD.

Sample Type	Vesicle Source	Marker(s)	Key Study Findings	Ref.
Plasma	Macrophage	Galectin 3	Enriched in MAFLD; Positively correlated with the severity of MASH; The highest correlation with MASH’s organizational gold standard	(49)
Plasma	Platelets	CD61	Enriched in MAFLD; increased with MAS	(49)
Plasma	Neutrophil	Ly-6G and Ly-6C	Enriched in MAFLD; The highest correlation with MASH’s organizational gold standard	(49)
Plasma	Hepatocyte	ASGR1 CYP2E1	Enriched in MAFLD; increased with MAS	(49)
Serum and plasma	Macrophage/monocyte	CD14	Enriched in MASH; Related to histological grading	(50)
Serum and plasma	invariant natural killer T cell	Vα24/Vβ11	Enriched in MASH; Related to MAS rating	(50)
Plasma and Hepatocyte	–	Haptoglobin VNN1 IGFALS	Enriched in steatotic hepatocytes (IMH cells treated with PA);	(54)
Plasma	–	FZD7	Enriched in MAFLD; Reduced after specific lifestyle interventions	(55)
Plasma	Liver	EpCAM CD133	Enriched in MAFLD; Significant increase in MASH compared to simple steatosis	(56)
Serum	Hepatocyte	SLC27A5 WISP1 AIMP1	The quantity and protein constituents of circulating EVs providing strong evidence for EV protein–based liquid biopsies for MAFLD/MASH diagnosis	(57)
Plasma	Hepatocyte	GLUT1	Early warning of MAFLD to distinguish the MAFL and MASH	(58)
Plasma	Liver	miR-122 miR-192	Enriched in MAFLD; Correlated with decreased liver expression;	(59)
Plasma	Liver	miR-122 miR-192 miR-128-3p	Biomarkers for MAFLD	(60)
Serum	–	miR-122	Early prediction of histological improvement of SGLT2I in MAFLD patients with diabetes after treatment	(61)
Plasma	–	miR-22-3p	A candidate biomarker for MAFLD patient stratification	(62)
Serum	–	miR-122-5p miR-335-5p miR-27a	Distinguished between simple obesity and MAFLD	(63)
Serum	–	miRNA122-5p, miRNA34a-5p, miRNA155-5p, miRNA146b-3p	Upregulated in circulating exosomes of children with MAFLD; Positively correlated with transaminases and uric acid.	(64)
Serum and ascitic fluid	–	miR-182 miR-301a miR-373	Potential biomarkers in MASH-induced liver cirrhosis with HCC	(65)
Urine	–	FFA (18:0) LPC (22:6/0:0) FFA (18:1) PI (16:0/18:1)	Potential biomarkers for distinguishing MASH and MAFL; Effectively reflects the degree and staging of liver fibrosis	(66)

ASGR1, Asialoglycoprotein receptor 1; CYP2E1, cytochrome P450 family 2 subfamily E member 1; Ly-6G and Ly-6C, common epitope on lymphocyte antigen 6 complex, locus G/C1; EpCAM, epithelial cell adhesion molecule; MAS, MAFLD activity score; VNN1, vanin 1; IGFALS, insulin-like growth factor-binding protein complex acid labile submit; SLC27A5, solute carrier family 27, member 5; WISP1, Wnt1-inducible signaling pathway protein-1; AIMP1, aminoacyl-tRNA synthetase interacting multifunctional protein 1; GLUT1, glucose transporter 1; FFA, Free fatty acid; LPC, Lysophosphatidylcholine; PI, Phosphatidylinositol; FZD7, Frizzled7; SGLT2, sodium glucose cotransporter 2 inhibitor; MASH, metabolic-associated steatohepatitis; MAFLD, metabolic-associated fatty liver disease.

### Circulating host-derived EV mi-RNAs as potential biomarkers for MAFLD

In addition to specific proteins, some miRNAs in liver-derived EVs could also be found in fatty liver disease and may act as non-invasive biomarkers for the disease. A previous study detected that circulating EVs in mice with MAFLD contained elevated levels of miR-122 and miR-192, which could act as indicators of liver injury (59). Furthermore, Newman et al. reported that miR-122, miR-192, and miR-128-3p in liver-specific ASGR1^+^ EVs could effectively discriminate between MAFL and MASH patients (60). Notably, serum exosomal miR-122 may also predict early histological improvements in MAFLD and diabetic patients treated with a Sodium Glucose Co-transporter 2 Inhibitor (SGLT2I) (61). Castaño’s team demonstrated that MAFLD patients with higher miR-22-3p level exhibit elevated fasting insulin and glucose levels, along with a less favorable lipid profile (62). Distinct exosomal miRNA expression profiles were also observed in children with MAFLD, with specific miRNAs such as miR-122-5p, miR-335-5p, miR-27a, and miR-34a-5p being upregulated (63, 64), implying the potential of certain EV miRNAs as serum markers for MAFLD in children. Individuals with MASH-related liver cirrhosis and HCC exhibited higher expression levels of miR-182, miR-301a, and miR-373 in exosomes found in the serum and ascitic fluid than those with MASH-related liver cirrhosis but without HCC, suggesting that exosomal miRNAs could also be used as early diagnostic indicators for HCC (65). Therefore, circulating miRNA in EVs could also serve as potential biomarkers for stratification and therapy monitor of MAFLD (as summarized in **Table 1**).

### Lipids in circulating host-derived EVs as potential biomarkers for MAFLD

**Figure 2:**
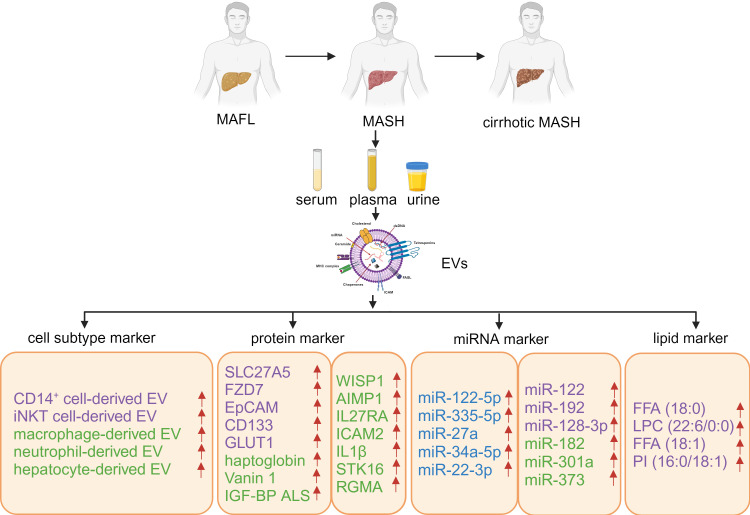
Host-derived EVs, containing specific proteins, miRNAs, and lipids, as biomarkers for MAFLD. The blue color indicates potential biomarkers for MAFL, the purple color represents potential biomarkers for the progression from MAFL to MASH, and the green color signifies potential biomarkers for MASH. Created with BioRender.com.

Besides proteins and miRNAs, lipids are another class of bioactive components in EVs to mediate metabolic changes in recipient cells. Zhu et al. explored MAFLD biomarkers from the lipidomic perspective of urinary EVs and discovered that the four lipid molecules, namely Free Fatty Acid (FFA) (18:0), Lysophosphatidylcholine (LPC) (22:6/0:0), FFA (18:1), and Phosphatidylinositol (PI) (16:0/18:1), could effectively distinguish between MASH from MAFL and reflect the degree and stage of liver fibrosis (66). Thus, lipids in EVs from the body fluids hold the promise to act as non-invasive biomarkers for MASH diagnosis and progression (as summarized in **Figure 2** and **Table 1**).

## Host-derived EVs as therapeutic agents for MAFLD

### Mesenchymal stem cells derived EVs as therapeutic agents for MAFLD

In addition to working as biomarkers, host-derived EVs can also hold promise as therapeutic agents for MAFLD. Stem cells are primarily derived from several distinct sources, including embryonic tissue; fetal tissues, such as the fetus, placenta (comprising the amniotic and chorionic membranes), amniotic fluid, and umbilical cord; as well as specific adult tissues, including adipose tissue, bone marrow, and blood (67). MSCs are multipotent progenitor cells with the potential for self-renewal and differentiation into mesenchymal lineages *in vitro* (68). Therapeutic applications of MSCs derived from various sources have been shown to be effective in treating neurological disorders, pulmonary dysfunction, metabolic and endocrine diseases, reproductive system disorders, skin burns, and cardiovascular diseases (68, 69). However, despite the safety demonstrated in preclinical and clinical studies, the long-term efficacy and safety of stem cell therapies necessitate validation through large-scale, randomized controlled Phase III clinical trials (68, 69). Notably, the therapeutic effects of these treatments are largely attributed to paracrine signaling rather than the prolonged survival and integration of transplanted cells (70). EVs can transfer bioactive molecules and facilitate intercellular communication, making them promising candidates for cell-free therapies (70, 71). Compared to stem cell treatments, EVs may provoke fewer immune responses, as they lack nuclei and most cellular organelles, thereby minimizing the risk of immune rejection (70, 71). Recent research has shown that miR-627-5p or Calcium/Calmodulin-Dependent Protein Kinase 1 (CAMKK1) enriched in human Umbilical Cord Mesenchymal Stem Cells (hUC-MSCs)-derived exosomes downregulates gluconeogenesis- and lipogenesis-related genes whereas upregulates fatty acid oxidation-related genes, thereby improving glucose and lipid metabolism *in vivo* and *in vitro* (72, 73). Specifically, the exosomal miR-627-5p downregulated the expression of Glucose-6-Phosphatase (G6Pc), Phosphoenolpyruvate Carboxykinase (PEPCK), Fatty Acid Synthase (FAS), Sterol Regulatory Element Binding Protein-1C (SREBP-1c) genes, and Fat Mass and Obesity-Associated Gene (FTO) in PA-induced L-O2 cells, whereas it upregulated the expression of Peroxisome Proliferator Activated Receptor α (PPAR-α). Studies have also shown that hUC-MSCs and their exosomes alleviate lipid deposition and liver fibrosis in MASH mice through mechanisms involving the upregulation of Etoposide-Induced gene 24 (EI24) protein and AMPK/mTOR signaling as well as activation of autophagy (74). Exosomes released by hUC-MSCs were also shown to mitigate hepatocellular inflammation in Methionine-Choline Deficient (MCD) diet-induced MASH mice by downregulating pro-inflammatory macrophages and cytokines such as Tumor Necrosis Factor alpha (TNF-α) and Interleukin 6 (IL-6), and facilitate the transition of macrophages into an anti-inflammatory state (75, 76). Moreover, hUC-MSC-derived exosomes exhibit antioxidation effects by activating the Nuclear factor erythroid-derived 2-like 2 (Nrf2)/NAD(P)H dehydrogenase, Quinone 1 (NQO-1) signaling pathway, which is crucial for MASH treatment (75).

In addition to hUC-MSCs, several other types of MSCs have demonstrated therapeutically potentials for MAFLD treatment. Human Amnion Mesenchymal Stem Cells (hAMSCs)-derived EVs have been shown to decrease the number of Kupffer Cells (KCs) and inhibit Hepatic Stellate Cell (HSC) activation in the livers of MASH rats and lower the mRNA expression of inflammatory cytokines such as TNF-α, IL-1, IL-6, and Transforming Growth Factor beta (TGF-β) (77). Small EVs (sEVs, 50-200 nm) from human Embryonic Stem Cell-derived Mesenchymal Stem/Stromal Cells (hESC-MSCs) also alleviate liver fibrosis in MASH mouse model (78). In a High Fat Diet (HFD)-induced MASH model, treatment with human Bone Marrow Mesenchymal Stem Cells (hBM-MSCs) or their derived exosomes (hBM-MSCs-Exo) upregulated miR-96-5p and downregulated caspase-2, leading to reduced expression of lipid synthesis (SREBP1/2, ACC) and uptake (CD36) genes. hBM-MSCs or hBM-MSCs-Exo also enhanced fatty acid oxidation genes, activated mitochondrial autophagy, and reduced apoptosis, contributing to MASH remission (79). Additionally, injection of human Adipose tissue-derived Stem Cells (hADSCs) or their sEVs increased anti-inflammatory macrophages in the livers of MASH model mice, thus improving inflammation and fibrosis (80). Zhao et al. reported that long-term HFD feeding extensively suppressed the expression of Brown Adipose Tissue (BAT)-associated genes such as Uncoupling Protein 1 (UCP1), and Peroxisome Proliferator-activated Receptor γ Coactivator α (PGC-1α) in epididymal White Adipose Tissue (WAT), while hADSCs significantly reversed these changes. Moreover, hADSC-derived exosomes reprogrammed adipose macrophages from the pro-inflammatory M1 to the anti-inflammatory M2 subtype, reducing WAT inflammation in HFD mice (81). Exosomes derived from human Liver Stem Cell (hLSC)-derived EVs enhanced liver functionality in MASH mice by downregulating genes associated with fibrosis and inflammation (82). Furthermore, exosomes obtained via intravenous administration of early Endothelial Progenitor Cells (EPCs) sourced from umbilical cord blood markedly improved hepatic steatosis, hepatocellular ballooning, and fibrosis, accompanied by decreased serum Alanine Transaminase (ALT) activity and MAS in Type 2 Diabetes Mellitus (T2DM) mice with stroke (83).

**Figure 3:**
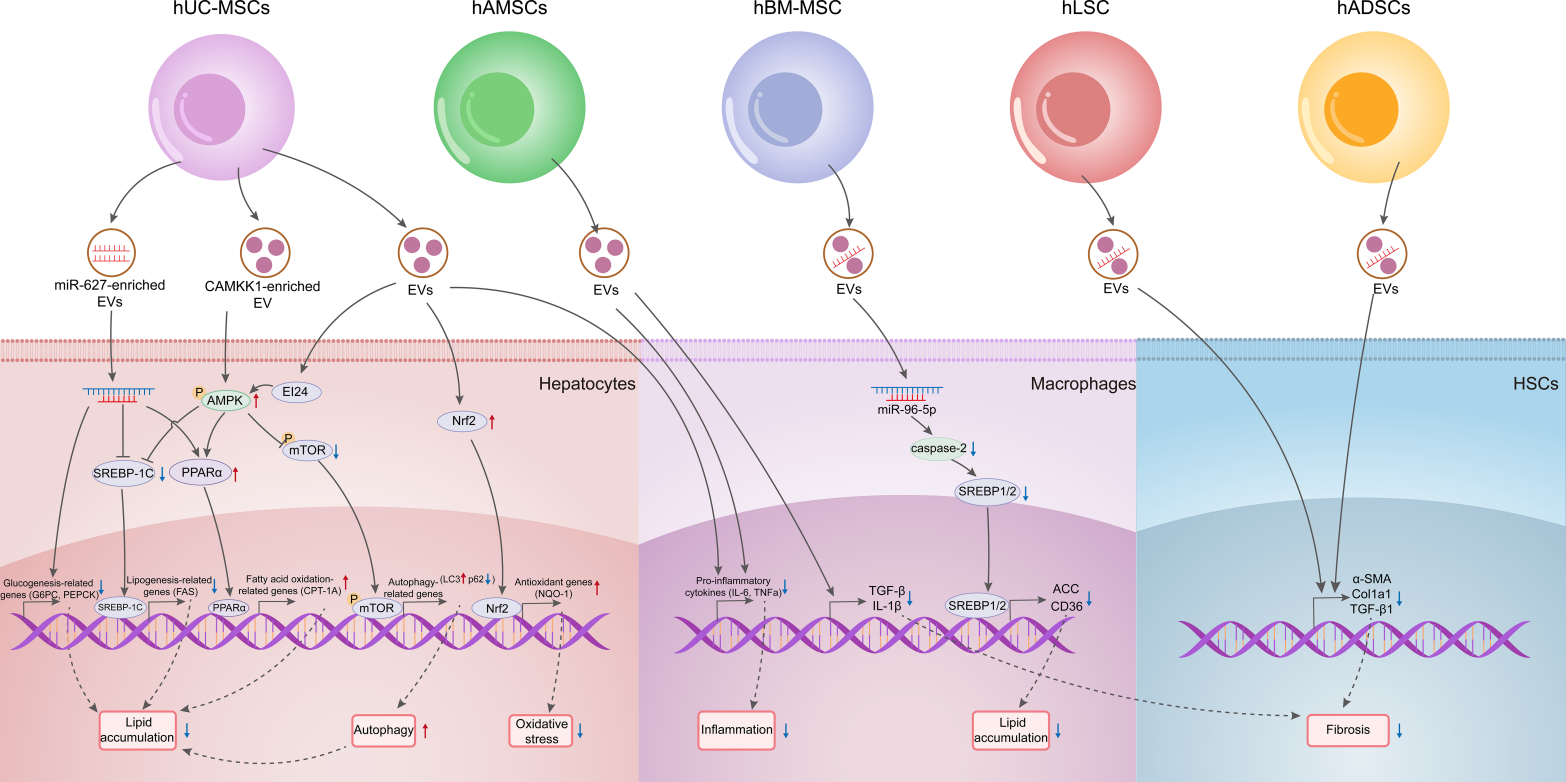
Therapeutic potentials of EVs derived from various types of MSCs on MAFLD.

Collectively, these findings indicate that MSC-derived EVs can mitigate lipid accumulation in liver cells by enhancing lipid oxidation, inhibiting lipid synthesis, and promoting autophagy, while also improving liver damage in MASH through anti-inflammatory and antioxidant effects. Thus, MSC-derived EVs exhibit considerable promise for treating MAFLD (as summarized in **Figure 3** and **Table 2**).

**Table 2:** Summary of the reported host-derived EVs as therapeutic agents for MAFLD.

Vesicle Type		Cell source		Molecular Mediators in the Vesicle		Recipient Targets Model		Function and Mechanism of Action	Ref.		
Exosomes		hUC-MSCs		miR-627-5p		MAFLD rat model (HFHFD), L-O2 cells (PA treatment)		Promoted cell viability and repressed apoptosis, Downregulated the expression of G6Pc, PEPCK, FAS, SREBP-1c and FTO and upregulated PPAR-α genes expression, Improves insulin sensitivity and liver injury in MAFLD rat	(72)		
Exosomes		hUC-MSCs		CAMKK1		MAFLD mice model (HFD), L-O2 cells (OA: PA mixture treatment)		Activate the AMPK signaling pathway, Upregulated PPAR-α/CPT-1A and downregulated SREBP-1c/FAS	(73)		
Exosomes		hUC-MSCs		–		MASH mice model (HFD and MCD), AML12 cells (OA: PA mixture treatment)		Alleviated lipid deposition and liver fibrosis, Upregulated EI24 protein and AMPK/mTOR signaling, Activated autophagy	(74)		
Exosomes		hUC-MSCs		–		MASH mice model (HFHCD/MCD), HepG2 cells (PA treatment), AML-12 cells (DMEM/F12 medium lacking methionine and choline treatment)		Altered the abnormal expression of lipid-related genes including SREBP-1c, PPAR-α, Fabp5, CPT1α, ACOX and FAS, Decreased the expression of F4/80^+^ macrophages, CD11c^+^ macrophages as well as the content of TNF-α and IL-6, Reduced the level of MDA and CYP2E1, Enhanced the protein ratio of p-Nrf2/Nrf2 and the protein expression of NQO-1	(75)		
Exosomes		hUC-MSCs		–		MASH mice model (MCD)		Reduced the level of TNF-α、IL-6、IL-1β, Rescued PPARα level, Increased the quantity of anti-inflammatory macrophages	(76)		
EVs		hAMSCs		–		MASH mice model (HFD), liver fibrosis rat model (CCl_4_ treatment)		Decreased the expression levels of TNF-α, IL-1β and IL-6, and TGF-β, Decreased fiber accumulation, KCs number, and HSCs activation, Suppressed the LPS/TLR4 signaling pathway	(77)		
sEVs (50–200 nm)		hESC-MSCs		–		MASH mice model (HFD)		Reduced serum IL-6 levels, Alleviated liver fibrosis	(78)		
Exosomes		hBM-MSCs		–		MASH mice model (HFD)		Upregulated miRNA-96-5p, PPAR-α, and CPT1 expression, Downregulated caspase-2, SREBP1, SREBP2, ACC, and CD36 expression, Reduced apoptosis, Activated mitochondrial mitophagy	(79)	
sEVs (50–200 nm)		hADSCs		–		MASH mice model (WD and LPS treatment)		Increased the number of anti-inflammatory macrophages in the liver, Improved liver inflammation and fibrosis	(80)		
Exosomes		hADSCs		STAT3		Obese mice model (HFD)	Enhanced the expression of BAT-related genes, such as UCP1, PGC1α, PRDM16, CIDEA, TBX1, and TMEM26, Reduced inflammation in WAT, Alleviated hepatic steatosis		(81)		
EVs		hLSCs		–		MASH mice model (MCD)	Reduced the expression levels of all fibrotic and pro-inflammatory genes, Inhibited liver inflammation and fibrosis		(82)		
Exosomes		human umbilical cord blood derived CD133^+^ cells		–		T2DM stroke mice model (photothrombotic ischemic stroke model)	Improved liver steatosis, hepatocellular ballooning, and fibrosis, Decreased serum ALT activity, and MAS, Improved neurological and cognitive function		(83)		
Exosomes		M2 macrophages		miR-411-5p		MASH mice model (HFHCD)	Inhibited HSCs activation by directly downregulating the expression of CAMSAP1		(84)		
Nanoparticles mimetic		Artificial liposomes		miR-690		MASH mice model (WD), liver spheroid system (primary hepatocytes, nonparenchymal cells and HSCs obtained from mouse livers with FAs, fructose, and LPS treatment)	Directly inhibited fibrogenesis in HSCs, inflammation in RHMs, and *de novo* lipogenesis in hepatocytes, Improved liver steatosis and fibrosis		(85)		
EVs		Neutrophils		miR-223		MASH mice model (HFD), AML12 cells (PA treatment)	Suppressed the expression of liver inflammation and fibrosis genes		(86)		
Exosomes		Macrophages		miR-223		MAFLD mice model (HFD), AML12 cells	Reduced profibrotic TAZ expression in hepatocytes, Attenuated liver fibrosis		(87)		

AML-12 cells, mouse immortalized hepatocytes; HepG2 cells, Human hepatocellular carcinoma cells; PA, palmitic acid; CYP2E1, cytochrome P450 2E1; PPAR-α, peroxisome proliferator activated receptor α; FASn/FAS, fatty acid synthase; SREBP-1c, sterol regulatory element binding protein-1C; TNF-α, tumor necrosis factor-α; IL-6, interleukin-6; MDA, malondialdehyde; CPT1α, carnitine palmitoyl transferase 1α; ACOX, acyl-coenzyme A oxidase; Fabp5, fatty acid-binding proteins 5; MASH, metabolic-associated steatohepatitis; IL-1β, Interleukin-1β; NQO-1, NAD(P)H dehydrogenase, quinone 1; Nrf2, nuclear factor erythroid derived 2 like 2; L-O2, Human normal liver cell line; G6PC, glucose-6-phosphatase; PEPCK, phosphoenolpyruvate carboxykinase; FTO, fat mass and obesity-associated gene; OA, oleic acid; AMPK, AMP-activated protein kinase; mTOR, mechanistic target of rapamycin; EI24, etoposide-induced 24; MAFLD, metabolic-associated fatty liver disease; HEK293T cells, human embryonic kidney cells; HFD, high-fat diet; CPT-1A, carnitine palmitoyltransferase 1A; CAMKK1, calcium/calmodulin-dependent protein kinase 1; CCl_4_, carbon tetrachloride; hAMSCs, human amnion-derived mesenchymal stem cells; hUC-MSCs, human umbilical cord mesenchymal stem cells; EVs, extracellular vesicles; TGF-β, transforming growth factor β; HSCs, hepatic stellate cells; KCs, Kupffer cells; LPS, lipopolysaccharide; TLR4, toll-like receptor 4; MSCs, mesenchymal stem cells; hESC, human embryonic stem cells; sEVs, small extracellular vesicles; BM-MSCs, bone marrow mesenchymal stem cells; BM-MSCs-Exo, bone marrow mesenchymal stem cells derived exosomes; SREBP-1, sterol regulatory element binding protein 1; SREBP-2, sterol regulatory element binding protein 2; ACC, acetyl coenzyme A carboxylase; CPT1, carnitine palmitoyltransferase 1; ALT, alanine transaminase; ADSCs, adipose-derived stem cells; STAT3, signal transducer and activator of transcription 3; BAT, brown adipose tissue; UCP1, uncoupling protein 1; PGC1α, proliferator-activated receptor gamma co-activator-1α; PRDM16, PR domain-containing 16; CIDEA, Cell death-inducing DNA fragmentation factor alpha-like effector A; TBX1, T-box transcription factor 1; TMEM26, transmembrane protein 26; WAT, white adipose tissue; LXR, liver X receptor; HLSCs, human liver stem cells; CAMSAP1, Calmodulin-Regulated Spectrin-Associated Protein 1; FAs, fatty acids; RHMs, recruited hepatic macrophages; AML12 cells, alpha mouse liver cells; TAZ, transcriptional activator with PDZ-binding motif; T2DM, type 2 diabetes mellitus; DM, diabetes mellitus.

### Immune cell-derived EVs as therapeutic agents for MAFLD

Since MALFD is also immunologically relevant, some immune cell-derived EVs can communicate with liver cells via certain miRNAs enriched in them, ameliorating liver fibrosis and inflammation in fatty liver mouse models. For instance, exosomal miR-411-5p from M2 macrophages has been shown to inhibit HSC activation in a MASH mouse model by targeting and suppressing the gene Calmodulin regulated Spectrin-Associated Protein 1 (CAMSAP1) (84). Furthermore, KCs can generate their own miR-690 and transfer it to liver cells, thereby recruiting hepatic macrophages, and HSCs via exosome release. Notably, miR-690 could directly suppress fibrogenesis, inflammatory responses, and *de novo* lipogenesis in HSCs, hepatic macrophages, and hepatocytes, respectively, improving liver steatosis and fibrosis in MASH mice (85). Additionally, in HFD-fed mice and PA-treated AML12 hepatocytes, hepatocytes could internalize miR-223-enriched EVs (primarily originating from neutrophils) via the interaction between the Low-Density Lipoprotein Receptor (LDLR) on hepatocytes and Apolipoprotein E (APOE) on the surface of neutrophil-derived EVs, suppressing liver inflammatory and fibrotic gene expression (86). Hou et al. discovered that HFD-fed mice with a myeloid-specific knockout of IL-6 receptor A (IL6Ra) showed reduced hepatic miR-223 expression and increased transcriptional activation of Tafazzin (TAZ), a miR-223 target gene, which exacerbated liver fibrosis. However, treatment with IL-6 prompts macrophages to secrete miR-223-enriched exosomes, reducing the expression of TAZ in liver cells via exosomal transfer, ultimately contributing to the attenuation of liver fibrosis in mice induced by HFD (87). Overall, immune cell-derived exosomes, enriched with specific miRNAs, play a crucial role in communicating with liver cells to alleviate liver fibrosis and inflammation in MASH (as summarized in **Table 2**).

## Host-derived EVs as drug delivery vehicles for MAFLD

Due to their self-derived nature and high compatibility with the host, host-derived EVs could be suitable drug carriers for MAFLD treatment (as summarized in **Table 3**). Research by Ding et al. demonstrated that inhibiting RNA-binding Protein J region (RBP-J) in myeloid cells reduces Notch signaling, leading to decreased inflammatory cytokine production and enhanced fatty acid metabolism in the livers of mice on a Choline Deficient Amino Acid (CDAA) diet, which ultimately alleviates liver inflammation in steatohepatitis models. They also found that intravenous administration of exosomes containing RBP-J decoy oligodeoxynucleotides effectively suppressed Notch signaling in hepatic macrophages, significantly improving MAFLD (88). Additionally, Kim’s study revealed that pan Peroxisome Proliferator-Activated Receptor (pan PPAR) agonists -primed MSC-derived EVs possess beneficial properties such as organ uptake, anti-steatotic effects, and tissue repair capabilities, which could greatly enhance MASH treatment (89). Therefore, using EVs as drug delivery carriers offers new opportunities for innovative MAFLD/MASH therapies.

**Table 3:** Summary of the reported host-derived EVs as drug delivery vehicles for MAFLD.

Drug	Vehicle	Research Model	Key Study Findings	Ref.
RBP-J decoy oligodeoxynucleotides	Exosomes derived from mouse endothelial cell line bEnd.3	MAFLD mice model (CDAA diet)	Myeloid-specific RBP-J deficiency up-regulated the expression of genes related to fatty acid degradation and peroxisomal fatty acid oxidation, decreased the expression of inflammatory factors IL1β and TNF-α, and attenuated experimental steatohepatitis in mice; Intravenous administration of exosomes containing RBP-J decoy oligodeoxynucleotides effectively suppressed Notch signaling in hepatic macrophages and improved MAFLD	(88)
pan PPAR agonists	EVs derived from MSCs	MASH mice model (MCD)	pan PPAR-primed MSC-derived EVs reduced steatotic changes and ameliorated ER stress and mitochondrial oxidative stress; Promoted liver regeneration via inhibiting apoptosis and enhancing proliferation	(89)

pan PPAR-iMSC-EVs, EVs from pan peroxisome proliferator-activated receptor agonist-primed induced mesenchymal stem cell; MASH, metabolic-associated steatohepatitis; ER, Endoplasmic reticulum; RBP-J, recombining binding protein for immunoglobulin Jkappa region; CDAA, choline deficient amino acid-defined diet; IL1β, interleukin-1 β; TNF-α, tumor necrosis factor α; MAFLD, metabolic-associated fatty liver disease.

## Host-derived EVs as therapeutic targets for MAFLD

### Hepatocytes-derived EVs as therapeutic targets for MAFLD

Hepatocytes constitute the main cell type in the liver, comprising approximately 80% of the total hepatic cell population (90). The primary contributing factor to MAFLD is overnutrition, which leads to the enlargement of adipose depots and the deposition of ectopic fat in the liver (5). Excessive lipid accumulation in hepatocytes leads to lipotoxicity, initiating a series of harmful events such as oxidative stress, inflammation, mitochondrial dysfunction, and ER stress, which eventually result in hepatocyte death (5). EVs released from lipotoxic hepatocytes mediate intercellular communication with target cells, such as macrophages, monocytes, and HSCs, thereby contributing to the pathogenesis of MAFLD.

Recent studies indicate that EVs released from lipotoxic hepatocytes communicate with macrophages to promote the liver’s inflammatory response and the progression of MAFLD. For example, in the MCD diet-induced MASH mouse model, exosomes enriched in miR-34a released by lipotoxic hepatocytes promotes liver inflammation by activating Kupffer cells (91). Similarly, exosomal miR-192-5p released by hepatocytes in rats with MASH activates pro-inflammatory macrophages via the Rictor/Akt/FoxO1 signaling pathway, which contributes to liver inflammation (92). Cholesterol-induced lysosomal dysfunction increases exosome release from Huh7 cells, which then polarizes pro-inflammatory M1 macrophages, thus triggering inflammation in a miR-122-5p-dependent manner (93).

PA can induce lipotoxicity in hepatocytes and is a widely used *in vitro* model for MAFLD. PA induces the release of lipotoxic EVs by activating Inositol Requiring Enzyme 1 alpha (IRE1α) (48). Activated IRE1α produces X-box binding protein 1 (XBP1), which further stimulates the expression of Serine Palmitoyltransferase (SPT) genes, thus initiating ceramide production and EVs secretion (94). Then lipotoxic EVs generate sphingosine-1-phosphate (S1P) with C16:0 ceramide as a substrate, thus activating macrophage chemotaxis and promoting hepatic inflammation (48, 94), which could be attenuated by inhibitors of sphingosine kinases, antagonists of S1P receptors (95), as well as the antagonist of S1P (96).

Mixed lineage kinase 3 (MLK3) was identified as another critical signaling molecule involved in the release of EVs from hepatocytes under lipotoxic conditions. PA or its metabolite LPC treatment induces the secretion of EVs enriched in the potent chemokine CXCL10 from Huh7 cells and primary mouse hepatocytes (PMHs) by activating the MLK3-STAT1 pathway (97), which in turn induce macrophage chemotaxis into the liver, leading to liver inflammation in FFC diet-induced MASH mice (98).

Notably, the Death Receptor 5 (DR5) pro-apoptotic signaling pathway is also implicated in lipotoxicity-induced release of EVs from hepatocytes. PA stimulates the DR5-Tumor Necrosis Factor Related Apoptosis Inducing Ligand (TRAIL)-caspase/Rho Associated Coiled-Coil Containing Protein Kinase 1 (ROCK1) apoptotic signaling pathway, leading hepatocytes to generate lipotoxic EVs, which in turn promote an inflammatory response in macrophages, while the ROCK1 inhibitor fasudil reduces serum levels of EVs and improves MASH (99).

Autophagy plays a significant role in the pathogenesis of MAFLD by regulating lipid metabolism, mitigating inflammation, and responding to cellular stress (100). Inhibition of autophagy through knockdown of autophagy-related gene 5 (ATG5) in murine hepatocytes AML12 cells resulted in the secretion of pro-inflammatory exosomes, enhancing Interleukin-1β (IL-1β) and TNFα co-treatment-induced liver inflammation and injury (101). These studies indicate that lipotoxic hepatocyte-derived EVs engage in close communication with macrophages, driving hepatic inflammation and disease progression.

HSCs play a key role in liver fibrosis, and recent studies have strongly linked the communication between HSCs and EVs derived from lipotoxic hepatocytes with the progression of MAFLD. In patients with MASH exhibiting F3-F4 fibrosis, activated HSCs and myofibroblasts exhibited a marked upregulation of the pro-fibrotic molecule, Cell Communication Network Factor 2 (CCN2), which subsequently potentiated HSC activation and aggravated liver fibrosis (102). *In vitro* studies indicated that lipotoxic hepatocytes-EVs induced by PA could be efficiently internalized by HSCs, leading to HSCs activation and subsequent liver fibrosis through inducing CCN2 (102). PA treatment also alters the miRNA expression profiles in hepatocyte-derived exosomes, which promotes HSCs activation by targeting PPAR-γ expression or Phosphatase and Tensin homolog (PTEN)-induced Kinase 1 (PINK1), thereby accelerating liver fibrosis progression (103–105). Besides PA, CoCl_2_-triggered chemical and intermittent hypoxia can also enhance the release of EVs from fatty acid-exposed HepG2 cells, triggering a pro-fibrotic response in human HSCs, and led to a higher release of circulating EVs in a CDAA diet-induced MASH model, thereby exacerbating hepatic fibrosis (106).

The lipotoxic EVs released by hepatocytes can also induce monocyte adhesion. In a mouse model of MASH induced by an FFC diet, integrin β1 (ITGβ1) present in lipotoxic EVs mediates monocyte adhesion to liver sinusoidal endothelial cells, thereby exacerbating hepatic inflammation (107). Notably, treatment with an ITGβ1 antibody significantly mitigates liver damage and fibrosis (107), indicating that it could be a promising therapeutic option to alleviate liver damage in MASH.

Considering the liver inflammation or HSC activation induced by EVs derived from lipotoxic hepatocytes, several pharmacological agents have been identified as potential treatments for MAFLD by reducing the release of EVs from these cells. Treatment of hepatocyte-derived EVs with ezetimibe modulates communication between hepatocytes and macrophages by inhibiting the NLRP3 inflammasome-IL-1β pathway, suggesting the potential of ezetimibe as a treatment for steatohepatitis (108). IL-22 therapy showed promise in MASH by mitigating the inflammatory effects of mitochondrial DNA-enriched hepatocyte-derived EVs (109). Inhibitors of Histone Deacetylase 2 (HDAC2) and DNA Methyltransferase 1 (DNMT1) reduced fibrogenic Th17 cell activity and fibrosis by downregulating angiocrine factors in hepatocytes-derived EVs (110). Elafin was found to upregulate miR-181b-5p and miR-219-5p in serum exosomes from livers of HFD-fed mice, which led to increased leptin expression in adipose tissue, alleviating hepatic steatosis (111). In addition, new-generation insulin-sensitizing thiazolidinediones or Mitochondrial Pyruvate Carrier 2 (MPC2) knockdown in hepatocytes inhibited HSC activation by reducing hepatocyte exosome secretion (112).

In summary, EVs from hepatocytes play a pivotal role in driving inflammation and fibrosis in fatty liver disease by activating macrophages/HSCs/monocytes and promoting inflammatory pathways (as summarized in **Table 4**). Therefore, they can be considered as therapeutic targets for MAFLD.

**Table 4:** Summary of the reported host-derived EVs as therapeutic targets for MAFLD.

Vesicle Type	Vesicle Source	Molecular Mediators in the Vesicle	Research Model	Key Study Findings	Ref.		
EVs	Hepatocyte	C16:0 ceramide	MASH mice mode (FFC diet); IMH cells (PA treatment)	PA induced C16:0 ceramide-enriched EVs release in an IRE1α-dependent manner; The ceramide metabolite, S1P, thus activating macrophage chemotaxis and promoting hepatic inflammation.	(48)		
Exosomes	Hepatocyte	miR-192-5p	MAFLD mice model (FFC diet)	Hepatocyte-derived exosomal miR-192-5p released by hepatocytes activated pro-inflammatory macrophages via the Rictor/Akt/FoxO1 signaling pathway, which contributes to liver inflammation.	(92)		
Exosomes	Hepatocyte	miR-122-5p	Huh7 and THP-1 cell lines (cholesterol treatment)	Cholesterol impairs hepatocyte lysosomal function, causing M1 polarization of macrophages via exosomal miR-122-5p.	(93)		
EVs	Hepatocyte	Ceramide	MASH mice model (FFC diet)	In mouse hepatocytes, activated IRE1α promotes transcription of SPT genes via XBP1, resulting in ceramide biosynthesis and release of EVs. These EVs recruited monocyte-derived macrophages to the liver, resulting in inflammation and injury in mice with diet-induced steatohepatitis.	(94)		
EVs	Hepatocytes	S1P	IMH cells (PA treatment)	PA-treated IMH release S1P-enriched EVs, which activate persistent and directional macrophage chemotaxis mediated by the S1P receptor, a potential therapeutic target for MASH.	(95)		
EVs	Plasma	S1P	MASH mice model (FFC diet)	The S1P antagonist ameliorates liver injury via inhibition of the production of S1P-rich steatotic EVs from hepatocytes.	(96)		
EVs	Hepatocyte	CXCL10	MASH mice model (FFC diet); PMH and Huh7 cells (PA or LPC treatment)	MLK3 induces the release of CXCL10-bearing EVs from hepatocytes, thereby promoting hepatic inflammation.	(97)		
EVs	Hepatocyte	TRAIL	MASH mice model (FFC diet), PMH and Huh7 cells (PA or LPC treatment)	Lipids, which stimulate DR5, induced release of hepatocyte EVs, which activate an inflammatory phenotype in macrophages.	(99)		
Exosomes	AML12 cells	HMGB1, HSP90	Atg5 knockout AML12 or mice (IL-1β/TNFα co-treatment)	Autophagy-deficient AML12 cells secreted exosomes with proinflammatory damage-associated molecular patterns, enhancing IL-1β and TNFα co-treatment-induced liver inflammation and injury.	(101)		
EVs	Hepatocyte	–	HepG2 cells (PA treatment)	PA-treated liver cells release EVs that induce upregulation of CCN2 expression in HSCs, which potentiates HSC activation and aggravates liver fibrosis.	(102)	
EVs	HepG2 cells, PMH	VNN1, miR128-3p	MASH mice model (MCD), MAFLD mice model (HFD), HepG2 cells, LX-2 cells, PMH, mHSCs	The hepatocyte-derived EVs stimulated by lipid toxic fatty acids are rich in VNN1 and miR128-3p. The former mediates the endocytosis of EVs by HSCs, while the latter inhibits the expression of PPAR-γ, activates HSCs, and thereby induces liver fibrosis.	(103)	
Exosomes	Hepatocyte	miRNA	Huh7 and HepG2 cells (PA treatment), LX-2 cells	Exosomes derived from PA-treated hepatocytes induce fibrotic activation of HSCs.	(104)	
Exosomes	L-O2 cells, serum	miR-27a	MAFLD mice model (HFD+CCl_4_)	Hepatocyte-derived exosomal miR-27a activated HSCs through the inhibition of PINK1-mediated mitophagy in MAFLD.	(105)	
EVs	Hepatocytes	–	MASH mice model (CADD diet), HepG2 cells (OA: PA mixture treatment)	CoCl_2_ treatment augmented release of EVs from fat-laden hepatocytes which induced a pro-fibrotic response in LX-2 cells; CoCl_2_ treatment also led to release of circulating EVs in a CDAA diet induced MASH model, promoting liver injury, inflammation and fibrosis.	(106)	
EVs	Hepatocyte	ITGβ1	MASH mice model (FFC diet), PMH (LPC treatment)	ITGβ1 is released from hepatocytes under lipotoxic stress as a cargo of EVs, and mediates monocyte adhesion to liver sinusoidal endothelial cells, which promotes hepatic inflammation.	(107)	
Exosomes	Hepatocyte	–	Mouse primary hepatocytes, human or primary mouse macrophages, HepG2 cells, MEF cells (PA treatment)	EVs isolated from hepatocytes treated with ezetimibe can regulate communication between liver cells and macrophages by inhibiting the NLRP3 inflammasome-IL1β pathway in macrophages, thereby inhibiting hepatic inflammation.	(108)	
EVs	Hepatocyte	mitochondrial DNA	MASH mice model (CXCL1 treatment and HFD)	IL-22 treatment weakens the inflammatory effects of mitochondrial DNA-enriched hepatocyte-derived EVs, thereby inhibiting liver inflammation in CXCL1-induced MASH.	(109)	
EVs	Plasma	IGFBP7, ADAMTS1	MASH minipig model (WD and CCl_4_ treatment)	Inhibitors of HDAC2 and DNMT1 reduced fibrogenic Th17 cell activity and fibrosis by downregulating angiocrine factors IGFBP7 and ADAMTS1 in hepatocytes-derived EVs.	(110)	
Exosomes	Serum	miR-181b-5p, miR-219-5p	MAFLD mice model (HFD)	The serum exosomes of Elafin-overexpressing HFD-treated mice showed an increase in miR-181b-5p and miR-219-5p, which can induce the expression of appetite reducing leptin in mouse mesenteric fat, thereby reducing obesity, hyperglycemia, and hepatic steatosis.	(111)	
Exosomes	Plasma	–	MASH mice model (FFC diet)	Insulin-sensitizing thiazolidinediones or hepatocyte MPC2 deletion inhibited HSC activation by reducing hepatocyte exosome secretion.	(112)	
Exosomes	Adipose tissue; plasma	–	Obese patients	Exosomes from adipose tissue of obese patients reduce insulin-stimulated Akt phosphorylation in human skeletal muscle cells and mouse hepatocytes, diminishing insulin sensitivity.	(115)	
EVs	Adipose tissue	–	HepG2 cells	EVs from adipose tissue of obese patients diminished insulin sensitivity in hepatocytes.	(116)	
ELVs	Adipose tissue	–	Obese mice model (HFD), C2C12 myocytes, mouse macrophages	ELVs from adipose tissue impairs glucose uptake and leads to insulin resistance.	(117)	
Exosomes	Adipose tissue	miR-103	MASH mice model (HFD), AML-12 cells (PA treatment)	Adipose tissue derived-exosome increased the levels of miR-103 in the liver which aggravates MASH by interacting with PTEN and inhibiting autophagy.	(119)	
EVs	Serum	miR-122, miR-192, miR-22	Alimentary obesity mice model (HFD)	Aerobic training changed the circulating EVs miRNA profile of obese mice, including decreases in miR-122, miR-192, and miR-22 levels, reduced the expression of PPAR-γ (a biomarker of adipogenesis) and liver steatosis score, thereby improving steatohepatitis in HFD mice.	(120)	
Exosomes	Adipocytes	Akr1b7	MASH mice model (HFD and MCD)	ATEx promotes hepatic steatosis to MASH by delivering exosomal Akr1b7 to hepatocytes, thus increasing the accumulation of glycerol and triglycerides in liver cells.	(122)	
Exosomes	Adipocyte	Resistin	Alimentary obesity mice model (HFD)	Melatonin decreased traffic volume of adipocyte-generated exosomal resistin from adipocytes to hepatocytes, which further alleviated ER stress-induced hepatic steatosis.	(123)	
Exosomes	VAT	–	HepG2 cells, HHSteC	Exosomes from VAT of obese patients integrated into liver cells and induced dysregulation of TGF-β pathway, contributing to the development of MAFLD.	(124)	
Exosomes	Intestine cells	PC	Obese mice model (HFD), murine hepatocytes, HepG2 cells, human monocytes, C57BL/6 murine colon epithelial, human embryonic kidney 293 cells, mouse-immortalized 3T3-L1 fibroblasts	Exosome-derived PC from HFD group binds to and activates AhR, leading to inhibition of the expression of genes essential for activation of the insulin signaling pathway, including IRS-2, and its downstream genes PI3K and Akt.	(131)
EVs	T Cells	–	LX-2 cells	EVs from activated and apoptotic T cells led to upregulation of MMP-1, MMP-3, MMP-9, and MMP-13 genes related to fibrinolysis in HSCs.	(132)
EVs	Adipose tissue macrophage	–	Obese mice model (HFD diet), PMH	EVs from adipose tissue macrophages in obese mice decreased Akt phosphorylation in primary hepatocytes, further impacting insulin sensitivity.	(133)

HFD, High-fat diet; S1P, sphingosine 1-phosphate; S1P_1_, sphingosine 1-phosphate receptor 1; CXCL1, C-X-C motif chemokine ligand 1; MASH, metabolic-associated steatohepatitis; IL-22, interleukin-22; BM-MSCs, bone marrow mesenchymal stem cells; CCl_4_, carbon tetrachloride; IGFBP7, insulin-like growth factor-binding protein 7; ADAMTS1, A disintegrin and metalloproteinase with thrombospondin motifs 1; HDAC2, histone deacetylase 2; DNMT1, DNA methyltransferase 1; MPC2, mitochondrial pyruvate carrier 2; EVs, extracellular vesicles; PPAR-γ, peroxisome proliferator-activated receptor gamma; CB1R, cannabinoid type 1 receptor; CB2R, cannabinoid type 2 receptor; CB, cannabinoids; MAFLD, metabolic-associated fatty liver disease; Akr1b7, aldo-keto-reductase 1B7; ATEx, endoplasmic reticulum stress-induced adipocyte-secreted exosome; Atg5, autophagy-related gene 5; HMGB1, high-mobility group box 1; HSP90, heat shock protein 90; AML12 cells, alpha mouse liver cells; HepG2 cells, human hepatoma cell line; HHSteC, human hepatic stellate cell line; TGF-β, transforming growth factor β; VAT, visceral adipose tissue; LX-2 cells, human hepatic stellate cells; CoCl2, cobalt chloride; L-O2, Human normal liver cell line; PINK1, phosphatase and tensin homolog (PTEN)-induced kinase 1; HSCs, hepatic stellate cells; CCN2, cell communication network factor 2; PA, palmitic acid; IMH, immortalized mouse hepatocyte; CXCL10, (C-X-C motif) ligand 10; PMH, primary mouse hepatocyte; LPC, ysophosphatidylcholine; MLK3, mixed lineage kinase 3; Rictor, Rapamycin-insensitive companion of mammalian target of rapamycin; Akt, protein kinase B; FoxO1, forkhead box transcription factor O1; IRE1α, inositol-requiring enzyme-1 alpha; XBP1, X-box binding protein 1; TRAIL, tumour necrosis factor-related apoptosis-inducing ligand; DR5, death receptor 5; ITGβ1, integrin β1; PC, phosphatidylcholine; PE, phosphatidylethanolamine; L-Exo, exosomes from lean animals; H-Exo, exosomes from obese animals; AhR, aryl hydrocarbon receptor; IRS-2, insulin receptor substrate 2; PI3K, phosphatidylinositol 3-kinase; PAI-1, Plasminogen activator inhibitor-1; SAAT, subcutaneous abdominal adipose tissue; AML-12, Alpha mouse liver 12 cells; Huh7, human HCC cell line; PTEN, phosphatase and tensin homolog; MEF cells, mouse embryonic fibroblast cells; IL1β, Interleukin-1β; NLRP3, NLR family pyrin domain containing 3; VNN1, vanin 1; mHSCs, primary mouse hepatic stellate cells; MMP, matrix metalloprotease; ELVs, exosome-like vesicles.

### Adipocytes-derived EVs as therapeutic targets for MAFLD

In addition to their role in secreting EVs, hepatocytes also act as recipient cells, taking up EVs from other tissues, including adipocytes, thus influencing disease progression. Metabolic disorders, such as obesity and insulin resistance, are acknowledged as significant risk factors for MAFLD (113, 114). For example, exosomes from subcutaneous abdominal adipose tissue (SAAT) of obese patients reduce insulin-stimulated Akt phosphorylation in human skeletal muscle cells and mouse hepatocytes, diminishing insulin sensitivity (115, 116). EVs enriched with IL-6, Monocyte Chemoattractant Protein-1 (MCP-1), and macrophage migration inhibitory factor (MIF), originating from the adipocytes of patients with aneurysmal aortic disease, have been shown to induce systemic insulin resistance and correlate positively with liver enzyme levels (116). Similarly, exosome-like vesicles (ELVs) from the adipose tissue of obese mice impair glucose uptake and promote insulin resistance both *in vivo* and *in vitro* (117). According to these studies, adipocyte-derived EVs play a significant role in inducing insulin resistance, which is pivotal in the pathogenesis of MAFLD.

EVs secreted by adipocytes can also promotes MAFLD by disturbing lipid metabolism in the liver. For example, adipocyte-derived exosomal LINC01705 increases lipid accumulation in high glucose-induced HepG2 cells via miR-552-3p/LXR axis modulation (118). In the MASH mouse model, adipose-derived exosomes enriched in miR-103 are taken up by hepatocytes, leading to autophagy inhibition and exacerbation of MASH, which could be reversed by miR-103 antagonist (119). Aerobic exercise has been shown to alleviate steatohepatitis in HFD-fed mice by downregulating specific miRNAs in WAT-derived EVs and improving liver steatosis scores (120). Aldo-keto reductase 1B7 (Akr1b7) is an enzyme that regulates lipid synthesis and plays a key role in hepatic lipid metabolism (121). Endoplasmic reticulum stress (ERS)-induced exosomes secreted by adipocytes promote the transition from liver steatosis to MASH by transporting exosomal Akr1b7 to hepatocytes (122). The adipokine resistin triggers liver steatosis by inducing ERS, while melatonin counteracted this effect by decreasing resistin delivery via adipocyte-derived exosomes (123). Exosomes derived from visceral adipose tissue (VAT) in obese patients can also be taken up by HSCs, disrupting the TGF-β pathway and contributing to the development of MAFLD (124). These studies indicate that adipocyte-derived EVs can carry various biomolecules that induce insulin resistance and lipid accumulation in hepatocytes, as well as disrupt the TGF-β signaling pathway, thereby influencing the onset and progression of MAFLD. Therefore, adipocyte-derived EVs may represent viable therapeutic targets for MAFLD.

### Intestine cell-derived EVs as therapeutic targets for MAFLD

The “gut-liver” axis plays a crucial role in the pathogenesis of MAFLD by regulating the gut microbiota, intestinal barrier, secreted signaling molecules, and immune responses, thereby influencing hepatic lipid metabolism and inflammatory responses (125, 126). Patients with MAFLD often suffer from gut microbiota dysbiosis, which disrupts the integrity of the intestinal barrier, resulting in increased permeability (127). This disruption of intestinal barrier function can lead to an increased release of pathogen-associated molecular patterns (PAMPs) like lipopolysaccharide (LPS) into the circulation and be transported to the liver where it actives toll-like receptor 4 (TLR4) and induces inflammation (128). As signaling molecules, EVs could mediates the crosstalk between the gut and the liver. The disruption of intestinal barrier function can lead to an increased release of EVs, and the composition of bioactive molecules they carry, such as LPS, lipids, proteins, and nucleic acids, will also change in MAFLD (129). Fizanne et al. reported that fecal EVs from MAFLD and MASH patients had a lower mean protein quantity and a greater content of LPS. Furthermore, MASH fecal EVs enhanced intestinal cell permeability and promoted the activation of LX2 cells (130). Research by Kumar et al. indicates that exosomes from the intestines of germ-free mice treated with antibiotics exhibit distinct lipid compositions between lean (L-Exo) and HFD-induced obese (H-Exo) groups, with L-Exo primarily containing phosphatidylethanolamine (PE) and H-Exo rich in phosphatidylcholine (PC). Specifically, the content of PC from the intestines of obese mice increases, whereas the content of PE content decreases (131). Analysis of fecal samples from five healthy volunteers and seven patients with Type 2 Diabetes (T2D) revealed that the number of EVs in the fecal samples of T2D patients was significantly higher (approximately 4.5×10^13^ particles/g feces) compared to those of healthy individuals (2×10^13^ particles/g feces). Additionally, the concentration of PC in EVs was also significantly increased (approximately 10%) in T2D patients, compared to that in healthy individuals, which was around 0.35% (131). This suggests that the diet-dependent increase in exosomal PC has clinical relevance. Intestine-derived EVs can be detected by biosensors in distant target tissues, such as Aryl Hydrocarbon Receptor (AhR). As a ligand-activated transcription factor, AhR can be activated by small molecules derived from diet, microbes, metabolism, and pollutants, playing complex regulatory roles in cellular physiology and pathology. When intestine-derived EVs are taken up by hepatocytes, the PC in H-Exo activates AhR as a ligand. The activated AhR then crosstalk with transcription factors to rewire hepatocyte metabolism and reprogram the cellular transcriptome. For instance, it inhibits the expression of key genes in the insulin signaling pathway, such as IRS-2, PI3K, and Akt, leading to insulin resistance. Additionally, the activation of AhR also affects the expression of fatty acid synthase and cholesterol synthase in the liver, altering the lipid composition of the liver. PC in H-Exo also activates AhR in adipocytes and skeletal muscle cells, inhibiting the expression of IRS-2 and further exacerbating insulin resistance. Moreover, H-Exo could be taken up by macrophages and promotes the expression of pro-inflammatory cytokines, such as TNF-α and IL-6, which further exacerbates liver inflammation (131). Overall, these findings suggest that intestine-derived EVs could serve as potential therapeutic targets for metabolic disease such as T2D and MAFLD.

### Immune cell-derived EVs as therapeutic targets for MAFLD

Immune cells also influence the progression of MAFLD by releasing EVs. Circulating microparticles (MPs) derived from activated and apoptotic T cells are increased in patients with active hepatitis C, which could be taken up by HSC in an ICAM-1-dependent manner and leads to activation of ERK1/2 and subsequent upregulation of fibrolytic matrix metalloprotease (MMP) genes and downregulation of procollagen α1(I) gene (132). In addition, exosomes from adipose tissue macrophages in obese mice decrease Akt phosphorylation and downstream GLUT4 in hepatocytes, and also suppress PPARγ in a miR-155 manner, finally impacting insulin sensitivity (133).

**Figure 4:**
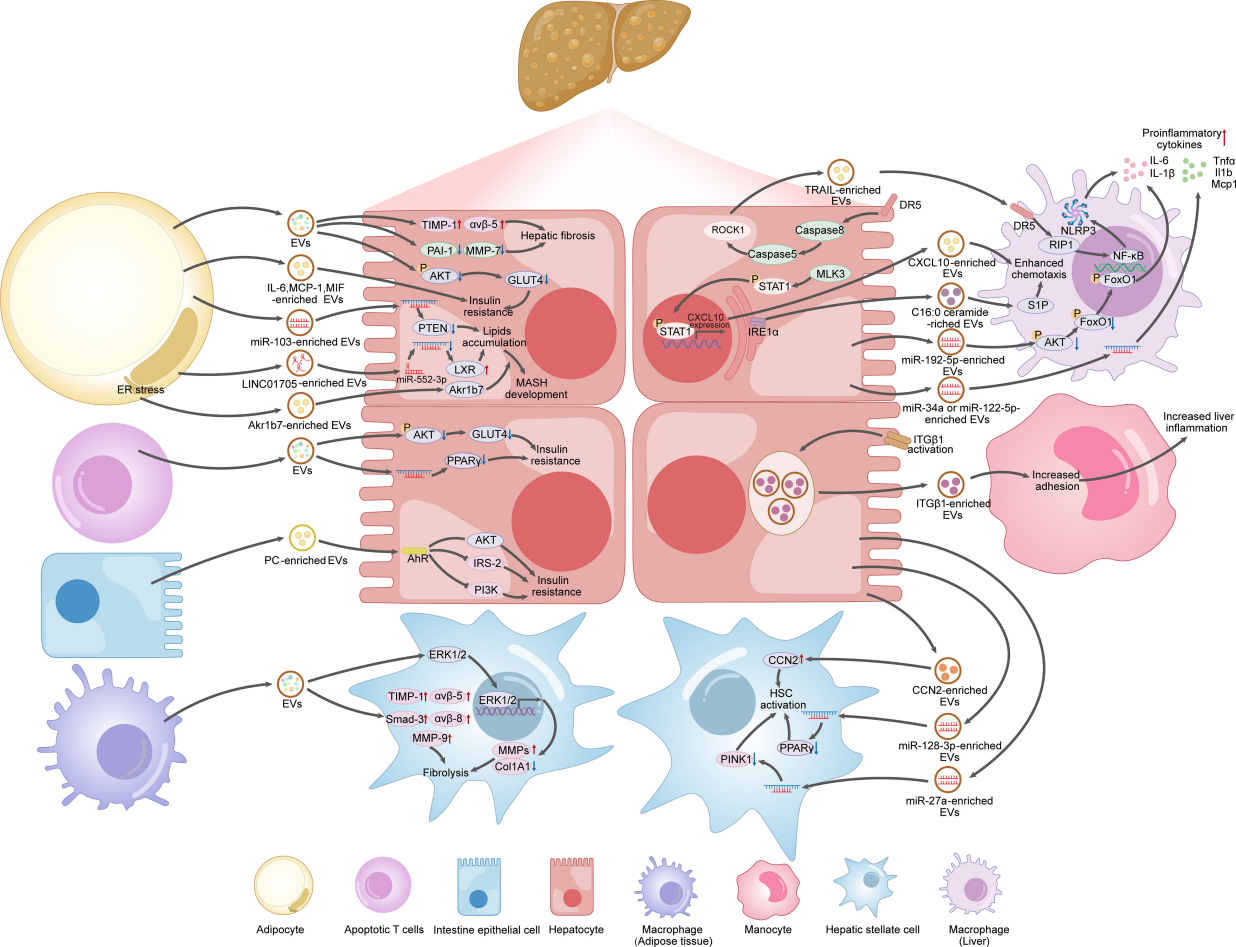
EVs produced by hepatocytes, adipocytes, intestine cells, immune cells, as potential therapeutic targets for MAFLD.

Taken together, EVs carry miRNAs, lipids and other biomolecules, allowing for communication between hepatocytes and other cell types and emerging as promising therapeutic targets for the management of MAFLD (as summarized in **Figure 4**).

## Plant-derived EVs as therapeutic agents for MAFLD

Current research primarily focuses on host-derived EVs, but studies on plant-derived EVs are also emerging. Halperin et al. first identified EVs from plant cells of carrots in 1967 (134). Plant-derived EVs influence intracellular physiological processes through their bioactive molecules, exhibiting pharmacological activities such as anti-inflammatory, antioxidant, anti-tumor, immune regulation, and promoting regeneration (135–137). Their excellent biocompatibility further underscores their potential as drug delivery systems, highlighting significant prospects in disease treatment (135–138). Recent findings suggest that plant-derived EVs can improve liver dysfunction in MAFLD by modulating key enzymes involved in fatty acid metabolism, glucose metabolism, and oxidative stress, indicating their potential as therapeutic agents for MAFLD. For example, Blueberry-derived Exosomes-Like Nanoparticles (BELNs) reduce oxidative stress in HepG2 cells by inhibiting Reactive Oxygen Species (ROS) generation and restoring mitochondrial membrane potential and improve insulin sensitivity in HFD-fed mice by downregulating fatty acid biosynthesis genes (139). Studies have demonstrated that Garlic-Derived Exosomes (GDE) effectively suppress inflammation in macrophages and improve liver dysfunction in HFD-fed mice, with miR-396e in GDE playing a crucial role in mediating interactions between macrophages and hepatocytes by targeting 6-phosphofructo-2-kinase/fructose-2,6-bisphosphatase 3 (PFKFB3) (140). In comparison to mice subjected to a high-fat high-sugar diet (HFHSD), Berger et al. found that genes encoding tight junction proteins Claudin-1 (CLDN1), Occludin (OCLN), and Zonula occludens-1 (ZO1) are upregulated in the jejunum of mice treated with orange juice-derived EVs, indicating that these EVs can enhance intestinal permeability in obesity (141). Ammonia borane is a hydrogen donor that can produce hydrogen with antioxidant, anti-inflammatory, anti-apoptotic, anti-fibrotic, and antimicrobial effects (142–144). Wang et al. discovered that oral administration of ginger-derived EVs loaded with ammonia borane alleviate insulin resistance and hepatic steatosis in a mouse model with T2DM accompanied by MAFLD (145). These studies suggest that plant-derived EVs can reduce liver lipid accumulation and inflammation, thereby alleviating liver dysfunction (as summarized **Table 5**). Additionally, plant-derived EVs can also improve intestinal barrier function and serve as carriers for drug delivery. These beneficial effects make them viable therapeutic agents for the treatment and prevention of MAFLD.

**Table 5:** Summary of the reported plant-derived EVs as therapeutic agents for MAFLD.

Vesicle Type	Source	Molecular Mediators in the Vesicle	Recipient Targets Model	Function and Mechanism of Action	Ref.
Exosomes-like nanoparticles	Blueberry	–	MAFLD mice model (HFD); HepG2 cells (rotenone treatment)	Reduced oxidative stress and cell apoptosis induced by rotenone in HepG2 cells; Accelerated the translocation of Nrf2 from the cytoplasm to the nucleus in rotenone-treated HepG2 cells; Reduced the expression of key genes related to fatty acid biosynthesis, FAS and ACC1; Improved insulin resistance and lipid droplet accumulation in HFD-fed mice.	(139)
Exosomes	Garlic	miR-396e	MAFLD mice model (HFD); THP-1 cells (LPS treatment); L-O2 cells	Inhibited inflammatory response and enhanced hepatic lipid metabolism through PFKFB3-mediated macrophage hepatocyte crosstalk.	(140)
Nanovesicles	Orange juice	–	Obese mice model (HFHSD)	Elevated expression of genes CLDN1, OCLN, and ZO1 encoding tight junction proteins to enhance intestinal permeability in obesity.	(141)

HepG2 cells, human hepatoma cell line; MAFLD, metabolic-associated fatty liver disease; Nrf2, nuclear factor erythroid 2-related factor 2; HFD, high-fat diet; FAS, fatty acid synthase; ACC1, acetyl CoA carboxylase 1; L-O2, Human normal liver cell line; GDE, garlic-derived exosomes; PFKFB3, 6-phosphofructo-2-kinase/fructose-2, 6-biphosphatase 3; LPS, lipopolysaccharide; ONVs, juice-derived nanovesicles; CLDN1, claudin-1; OCLN, occludin; ZO1, zonula occludens-1.

## Microbial-derived EVs as therapeutic targets for MAFLD

Emerging evidence underscores the critical role of intestinal microbiota dysbiosis in the development of metabolic disorders, including obesity, type 2 diabetes, and MAFLD (146, 147). As these diseases progress, disruption of the intestinal barrier often facilitates the translocation of microbial metabolites into the bloodstream, affecting distant organs (146, 148). Besides metabolites, microbial DNA is also found to be enriched in the circulation and linked to metabolic disorders and tissue inflammation in obese individuals and animal models (149–151). Research demonstrated that the bacterial DNA originates from their EVs (152). mEVs were first identified by Work et al. in 1966 in *Escherichia coli* (153). Subsequent studies observed the production and release of mEVs from a diverse range of microbial species in the host circulation (154, 155). mEVs are generated from the outer membrane of bacteria and play a significant role in host-microbe communication (156). They perform various functions in host interactions, including transporting virulence factors that enhance pathogenicity and facilitate immune evasion, transferring DNA to influence host gene expression, carrying nutrient-related proteins for resource acquisition in nutrient-limited environments, facilitating intra- and interspecies communication among bacteria, and enabling rapid membrane remodeling for environmental adaptation (157). Furthermore, mEVs and host cell-derived EVs exhibit distinct surface markers (157, 158). Host-derived EVs are characterized by specific markers such as CD63, CD81, and CD9, while mEVs contain unique outer membrane proteins, including OprO, OprF, and OprB (157, 158). During the onset of metabolic diseases, impaired intestinal barrier facilitates the leakage of mEVs into the circulation, allowing them to communicate with target organs of the host (159–161). Recent research indicates a link between mEVs and MAFLD, suggesting their potential as therapeutic targets for MAFLD (as summarized in **Table 6**). Intestinal mEVs derived from HFD-fed mice, particularly those originating from *Pseudomonas panacis*, could readily cross the intestinal barrier and be taken up by insulin-responsive tissues such as the liver, adipose tissue, and skeletal muscle, disrupting insulin signaling and inducing insulin resistance (160). mEVs from MAFLD mice can also deliver microbial DNA to pancreatic β cells, hepatocytes, and HSCs, leading to impaired insulin secretion, enhanced hepatic inflammation, and exacerbation of liver fibrosis through the activation of the cGAS/STING pathway (152, 162, 163). The clearance of microbials and mEVs from circulation relies on the V-set and immunoglobulin domain containing 4 positive (Vsig4^+^) or Complement receptor of the immunoglobulin superfamily positive (CRIg^+^) macrophages in the liver through a C3-dependent opsonization mechanism (152, 162, 163). However, the proportion of these cell populations decreases during the development of MAFLD/MASH, leading to microbial DNA accumulation and subsequent detrimental effects in target cells (152, 162, 163).

**Table 6:** Summary of the reported microbial-derived EVs as therapeutic targets for MAFLD.

Vesicle Type	Source	Molecular Mediators in the Vesicle	Recipient Targets Model	Key Study Findings	Ref.
EVs	Gut microbe	DNA	Obese mice model (HFD), hepatocyte, adipocyte	Depletion of CRIg^+^ cells results in the spread of mEVs into distant metabolic tissues, subsequently exacerbating tissue inflammation and metabolic disorders; treatment of obese mEVs directly triggers inflammation and insulin resistance of hepatocytes and adipocytes.	(152)
EVs	Stool	–	HFD-fed mice	*In vivo* administration of EVs from HFD-fed mice induced insulin resistance and glucose intolerance compared to RD-fed mice; Pseudomonas panacis-derived EVs blocked the insulin signaling pathway in both skeletal muscle and adipose tissue.	(160)
EVs	Gut microbe	DNA	Obese mice model (HFD)	mEVs readily pass through obese gut barrier and deliver microbial DNAs into β cells, resulting in elevated inflammation and impaired insulin secretion by triggering cGAS/STING activation.	(162)
EVs	Gut microbe	DNA	MASH mice model (WD), Vsig4^-/-^mice model	In the absence of Vsig4^+^ macrophages, gut mEVs translocation led to microbial DNA accumulation in hepatocytes and HSCs, resulting elevated hepatocyte inflammation and HSCs fibrogenic activation; Vsig4^-/-^ mice more quickly developed significant liver steatosis and fibrosis than WT mice after WD feeding.	(163)
Exosomes	Serum	HMGB1	HFD-fed mice	Exosomes in the intestine of HFD mice enhanced the release of HMGB1, which can be transported from the intestine to the liver through exosomes, leading to hepatic steatosis in ecological disorders.	(164)
EVs	*Akkermansia muciniphila*	–	Liver injury mice model (HFD and CCl_4_ treatment), LX-2 cells (LPS treatment)	EVs treatment inhibited the expression of TLR-2 and TLR-4 genes in LPS stimulated LX-2 cells; EVs inhibited the activation of HSC in HFD and CCl_4_ induced liver fibrosis mice and significantly reduced the expression of fibrosis and inflammation biomarkers in mouse tissues	(167)

EVs, extracellular vesicles; mEVs, DNA-containing extracellular vesicles; cGAS, cyclic GMP-AMP synthase; STING, cyclic GMP-AMP receptor stimulator of interferon genes; HFD, high-fat diet; RD, regular diet; HMGB1, high mobility group box 1; ASC, apoptosis-associated speck-like protein containing a CARD; OMVs, outer membrane vesicles; LX-2 cells, human hepatic stellate cell line; MASH-fEVs, extracellular vesicles isolated from the feces of MASH patients; MASH, metabolic-associated steatohepatitis; WT, wild-type; HSCs, hepatic stellate cells; LPS, lipopolysaccharide; TLR-4, toll-like receptor 4; TLR-2, toll-like receptor 2.

In HFD-induced MAFLD mouse models with disrupted gut microbiota, levels of the pro-inflammatory cytokine high mobility group box 1 (HMGB1) are significantly elevated in the intestinal tissue, enabling its transfer via mEVs from the compromised intestinal barrier to the liver, where it activates Toll-like Receptor 4 (TLR4), ultimately resulting in hepatic inflammation and liver dysfunction (164, 165). However, treatment of LPS-stimulated HSCs with both live and pasteurized *Akkermansia muciniphila*, a symbiotic bacterium that colonizes the intestinal mucosa and improves intestinal health and metabolic status (166), along with its EVs, significantly reduced the expression of TLR-2 and TLR-4 genes, and ameliorated liver inflammation and fibrosis, with EVs showing the most effective recovery of HSCs (167). This research highlights a novel approach to treating liver inflammation and fibrosis using EVs from specific probiotics.

**Figure 5:**
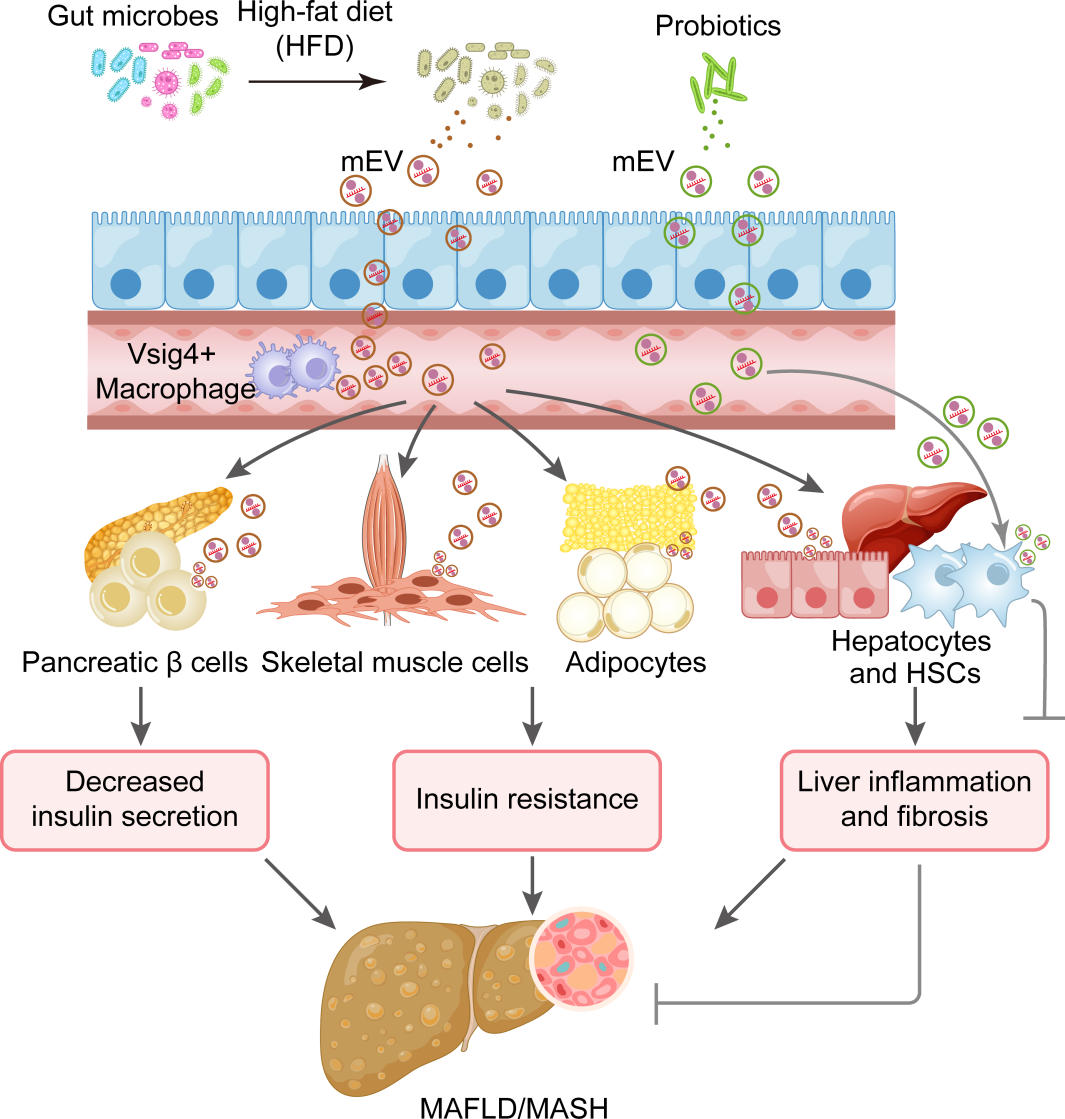
EVs produced by microbials as therapeutic targets for MAFLD.

In summary, MAFLD is characterized by compromised intestinal barrier and a significant reduction in the population of Vsig4^+^ and CRIg^+^ macrophages, which allows mEVs to transport bacterial DNA or pro-inflammatory cytokines to distant target cells, including hepatocytes, HSCs, adipocytes, pancreatic β cells, and skeletal muscle cells, thereby leading to insulin resistance, liver inflammation and dysfunction. Notably, certain probiotic-derived mEVs exhibit protective effects against this condition. Therefore, mEVs present promising therapeutic targets for the treatment of MAFLD (as summarized in **Figure 5**).

## Conclusions and future perspectives

As an important part of nanomedicines, EVs transport a diverse array of cellular cargo, including proteins, lipids, and nucleic acids, which could be internalized by target cells. This internalization facilitates the transfer of bioinformation that reflects the condition of the donor cell to the recipient cell, occurring in both healthy and pathological contexts, such as liver disease. Recent review articles have emphasized the critical roles of EVs in the pathogenesis of MAFLD and MASH (168–172), underscoring their potential as biomarkers (168, 171–173), therapeutic targets (170), and innovative treatment strategies (168, 171, 172). Lu et al. summarized the contribution of EVs to the pathogenesis of alcoholic fatty liver disease and MAFLD (169). Jiang et al. explored the mechanistic pathways through which EVs influence various biological processes, such as metabolic dysregulation, immune dysfunction, gut microbial imbalances, and fibrotic progression in MAFLD, highlighting their promise as non-invasive biomarkers and therapeutic modalities (168). Wang et al. examined the role of EVs in MASH pathogenesis, providing evidence for the therapeutic potential of stem cell-derived EVs and their use as clinical biomarkers for diagnosis, staging, and prognosis (171). Zhu et al. focused on the biological functions and underlying mechanisms of EVs in fibrotic diseases, further delineating their role as both biomarkers and therapeutic agents (172). Garcia et al. proposed a strategy to identify EV surface proteins as diagnostic biomarkers for MAFLD (173). Wu et al. discussed the origin, characteristics, cargo, and functional roles of EVs within the context of MAFLD, emphasizing their potential as novel therapeutic targets (170). This review is the first to comprehensively explore the diverse functions of EVs from hosts, plants, and microbes in MAFLD, offering a detailed synthesis of current evidence. Notably, we underscored the substantial potential of host-derived EVs as non-invasive biomarkers, therapeutic agents, therapeutic targets, and drug delivery systems for MAFLD. Additionally, we discussed the emerging therapeutic applications of plant-derived EVs in MAFLD prevention and treatment, as well as the promise of microbiota-derived EVs as an innovative therapeutic approach for MAFLD. Mechanistically, host-derived EVs influence the progression of MAFLD by mediating intercellular communication through bioactive molecules carried from the parental cells under disease conditions. Therefore, targeting these EVs presents a potential strategy for intervening in disease progression. EVs derived from MSCs and immune cells can also facilitate intercellular communication through their cargo, primarily alleviating MAFLD by modulating lipid metabolism, reducing inflammation, and mitigating fibrosis. Plant-derived EVs not only alleviate hepatic lipid accumulation and inflammation but also reduce MAFLD severity by enhancing intestinal barrier function. Furthermore, microbial-derived EVs contribute to the progression of MAFLD by inducing insulin resistance, liver inflammation, and liver dysfunction. EVs derived from these three distinct sources exert their effects on MAFLD through different mechanisms, which may be attributed to differences in their composition, including both the cargo and surface markers. The physiological and pathological states of the originating cells may also influence the content and biomarkers of the EVs produced (158). Given the variation in the expression of cell surface receptors by EVs, their effects on recipient cells may also differ (158). Furthermore, this heterogeneity may be influenced by the originating organ or tissue of the EVs (174). For example, host-derived EVs are characterized by molecular markers such as CD63, CD81, and CD9 (158), whereas microbial-derived EVs contain unique outer membrane proteins such as OprO, OprF, and OprB (157).

Despite the growing interest in EVs as biomarkers and therapeutic agents, their clinical application remains challenging due to several key limitations. First, current methods for EV isolation, capture, and analysis are not standardized, making it difficult to ensure the purity, activity, and stability of EVs across different cell types (175, 176). There is a critical need for protocols that adhere to good manufacturing practices to ensure product quality and cost-efficiency, as existing methods can be time-consuming or expensive (177). Another issue concerns the low delivery efficiency of the produced EVs. For instance, intravenously injected EVs are prone to clearance by the mononuclear phagocyte system (42), which calls for the development of liver-targeting strategies, such as integrin modification. Safety is another concern, particularly regarding the immunogenicity of microbial-derived EVs, which may activate TLR pathways and require thorough safety evaluations. Furthermore, regulatory hurdles persist, as there is no standardized process for EV production or clear guidelines for assessing their safety (42, 178). Given these challenges, improved collaboration is needed between EV researchers, nanomedicine experts, regulatory bodies, and clinical institutions to advance the clinical translation of EVs. Due to challenges such as the purity of isolated EVs, production scalability, and targeted delivery, the lack of clinical trials remains a significant limitation in the application of EVs. Nonetheless, the body of related research is continuously growing, providing a foundation for the future clinical application of EVs in MAFLD management.

## Glossary

AhR: Aryl hydrocarbon receptor
Akr1b7: Aldo-keto-reductase 1B7
APOE: Apolipoprotein E
ASGR1: Asialoglycoprotein Receptor 1
Atg5: Autophagy-related 5
BELNs: Blueberry-derived exosomes-like nanoparticles
BAT: Brown Adipose Tissue
CAMKK1: Calcium/Calmodulin-Dependent Protein Kinase 1
CCN2: Cell Communication Network Factor 2
CDAA: Choline Deficient Amino Acid
DNMT1: DNA Methyltransferase 1
ECM: Extracellular Matrix
ELVs: Exosome-Like Vesicles
EPCs: Endothelial Progenitor Cells
ERS: Endoplasmic Reticulum Stress
EVs: Extracellular Vesicles
FAS: Fatty Acid Synthase
FFA: Free Fatty Acid
FFC: Fat, Fructose, and Cholesterol
FTO: Fat Mass and Obesity-Associated Gene
GDE: Garlic-Derived Exosomes
GLUT1: Glucose Transporter 1
G6Pc: Glucose-6-Phosphatase
HCC: Hepatocellular Carcinoma
HDAC2: Histone Deacetylase 2
HFD: High Fat Diet
hAMSCs: Human Amnion Mesenchymal Stem Cells
hESC-MSCs: Human Embryonic Stem Cell-derived Mesenchymal Stem/Stromal cells
hADSCs: Human Adipose Tissue-derived Stem Cells
hBM-MSCs: Human Bone Marrow Mesenchymal Stem Cells
hLSC: Human Liver Stem Cell
HMGB1: High-Mobility Group Box 1
HSC: Hepatic Stellate Cell
hUC-MSCs: human Umbilical Cord MSCs
IGF-BP ALS: Insulin-Like Growth Factor-Binding Protein Complex Acid Labile Subunit
IL-1β: Interleukin-1β
IL6Ra: IL-6 receptor A
iNKT: invariant Natural Killer T
ITGβ1: Integrin β1
KCs: Kupffer Cells
LDLR: Low-Density Lipoprotein Receptor
LPC: Lipopolysaccharide
LPS: Lysophosphatidylcholine
MAFLD: Metabolic-Associated Fatty Liver Disease
MCD: Methionine-Choline Deficient
MCP-1: Monocyte Chemoattractant Protein-1
MMP: Matrix Metalloproteinase
MPs: Microparticles
MPC2: Mitochondrial Pyruvate Carrier 2
MSCs: Mesenchymal Stem Cells
NAFLD: Non-Alcoholic Fatty Liver Disease
MAS: MAFLD Activity Score
MASH: Metabolic-Associated Steatohepatitis
PA: Palmitic Acid
pan PPAR: pan Peroxisome Proliferator-Activated Receptor
PE: Phosphatidylethanolamine
PEPCK: Phosphoenolpyruvate Carboxykinase
PMHs: Primary Mouse Hepatocytes
PPAR-α: Peroxisome Proliferator Activated Receptor α
ROCK1: Rho Associated Coiled-Coil Containing Protein Kinase 1
ROS: Reactive Oxygen Species
SAAT: Subcutaneous Abdominal Adipose Tissue
SGLT2I: Sodium Glucose Co-transporter 2 Inhibitor
SREBP-1c: Sterol Regulatory Element Binding Protein-1C
S1P: Sphingosine 1-Phosphate
TAZ: Transcriptional Activation with PDZ-binding Motif
TRAIL: Tumor Necrosis Factor Related Apoptosis Inducing Ligand
T2DM: Type 2 Diabetes Mellitus
T2D: Type 2 Diabetes
UCP1: Uncoupling Protein 1
Vanin 1: Vascular Non-Inflammatory Molecule-1
VAT: Visceral Adipose Tissue
WAT: White Adipose Tissue
